# Iatrogenic rupture of the left main bronchus secondary to repeated surgical lobe torsion during double-lumen tube placement: A case report

**DOI:** 10.1097/MD.0000000000007694

**Published:** 2017-08-04

**Authors:** Zong Ming Jiang, Chu Zhang, Zhong Hua Chen

**Affiliations:** a Department of Anesthesia; b Department of Thoracic surgery, Shaoxing People's Hospital (Shaoxing Hospital of Zhejiang University), Shaoxing, P.R. China.

**Keywords:** bronchial rupture, double lumen tube, surgery

## Abstract

**Rationale:**

Bronchial rupture is a rare but potentially life-threatening complication during double-lumen endobronchial tube placement. The rupture of the left main bronchus resulting from repeated surgical torsion is uncommon.

**Patient concerns:**

A 70-year-old man with a history of chronic obstructive pulmonary disease (COPD), intermediate emphysema, chronic bronchitis, hypertension, type 2 diabetes mellitus, and L3-L4 lumbar intervertebral disc herniation. Chest x-ray and computed tomography revealed a solitary pulmonary nodule in the left lower lobe.

**Diagnoses:**

Left lower lobe carcinoma.

**Interventions:**

To improve surgical access, forceps were used to oppress and torque the left lung.

**Outcomes:**

An irregular, circular, horizontal, full-thickness rupture of 1.2 cm was observed at the tip of the bronchial tube in the left main bronchus upon examination of the bronchial stump.

The rupture was repaired via primary suturing with 4–0 prolene thread and secondary reinforcement with a pericardial flap through a left thoracotomy, with no further complications.

**Lessons:**

Caution should be exercised during compression and torsion of the pulmonary lobe when attempting to improve surgical access, especially in patients with COPD. Conversion to thoracotomy is recommended if other measures have been unsuccessful.

## 1 Introduction

Placement of a double-lumen tube is frequently performed to ensure lung isolation during thoracic surgery. Tracheobronchial injuries, whether from iatrogenic or traumatic causes, can be challenging to manage and sometimes life threatening. Risk factors for airway injury include inexperienced anesthesiologists, repeated attempts to intubate, tracheobronchial malformation, overexpansion of the balloon cuff, and advanced age.^[1–3]^ The reported incidence of airway injury varies from 0.05% to 0.2% in previous studies.^[4–6]^ A total of 88 cases involving double-lumen endobronchial tube-related airway injury were reported between 1972 and 2010.^[5,7]^ However, bronchial injury due to surgical manipulation is an extremely uncommon and seldom-reported complication, especially following repeated lobe torsion during placement of an indwelling double-lumen tube. In the present report, we discuss the case of a patient who experienced left main bronchus rupture after several surgical lobe torsions for improving access and vision.

## 2 Case description

The patient of the present case was a 70-year-old man (height: 170 cm; weight: 66 kg) with a history of chronic obstructive pulmonary disease (COPD), intermediate emphysema, chronic bronchitis, hypertension, type 2 diabetes mellitus, and L3-L4 lumbar intervertebral disc herniation. Chest x-ray during routine physical examination revealed a solitary pulmonary nodule in the left lower lobe. Computed tomography (CT) confirmed a 1.5 × 1.2 × 2.4 cm solid nodule in the left lower lobe, which became larger after 3 months.

His medications included tiotropium and salbutamol nebulization for COPD, amlodipine besylate and losartan for hypertension, and acarbose and repaglinide tablets for diabetes mellitus. Preoperative pulmonary function tests revealed a forced expiratory volume in 1 second of 1.25 L, whereas the ratio of forced expiratory volume in 1 second to forced vital capacity was 55%. The diffuse capacity of the lung for carbon monoxide was 12.3 mL/min/mm Hg (72% of predicted value). His preoperative vital signs were as follows: blood pressure, 135/80 mm Hg; heart rate, 62 beats/minute; temperature, 36.5°C; and respiratory rate, 18–20 beat/min. Modified Mallampati airway classification was grade II. The patient exhibited a moderate barrel chest and diminished breath sounds, although no cardiac murmurs were observed.

Electrocardiography, pulse oximetry, blood pressure measurements, and end-tidal carbon dioxide waveforms were evaluated prior to surgery. A cannula was inserted into the right radial artery. Preoxygenation (6 L/min) was performed via a disposable face mask. General anesthesia was induced via an intravenous injection of midazolam, lidocaine, propofol, sufentanil, and rocuronium. The patient was intubated with a left-sided 37-French, Robershaw double-lumen tube using a stylet. The stylet was removed immediately after the tip of the tube had passed beyond the vocal cords. The tube was advanced by rotating counterclockwise at 90 degrees. The tube was inserted smoothly, and no resistance was encountered at any time during placement. Proper bronchial tube position was confirmed through a fiberoptic bronchoscope (FOB). Satisfactory positioning was attained with the proximal edge of the bronchial cuff at least 0.5 cm below the carina in the left bronchus. The left bronchial cuff was inflated with 2 mL of air, whereas the tracheal cuff was inflated with 6 mL of air. Cuff pressure was not measured during the procedure. The patient was then transferred to the right lateral decubitus position. During this change in position, the bronchial cuff was completely deflated, whereas the tracheal cuff was inflated. The FOB was used to reconfirm bronchial tube position following the change in posture. No bleeding or secretions were observed, and satisfactory positioning was confirmed. Anesthesia was maintained using propofol (effect site concentration: 2.5 to 4.0 μg/mL) and remifentanil (effect site concentration: 4.5 to 6.0 ng/mL) under target-controlled infusion. A bispectral index monitoring system (Aspect Medical System, MA) was utilized to maintain an anesthesia depth between 50 and 60.

Left lower lobectomy was performed via video-assisted thoracoscopy (VATS). When the thoracoscope entered the thoracic cavity, the surgeon observed that the left lung was not fully collapsed due to concurrent COPD-decreased lung recoil and emphysema, whereas the right lung was normally ventilated. The nondependent lung was suctioned. Observation through the inserted FOB revealed that the bronchial tube was in the appropriate place. Following consultation with experts in thoracic surgery, carbon dioxide was insufflated into the left cavity via the thoracoscope to compress the left lung and improve surgical access, whereas the left bronchus was simultaneously suctioned using a suction catheter. However, this intervention only partially collapsed the aerated left alveolar tissue. To further improve surgical access, forceps were used to oppress and torque the left lung. After several attempts, a relatively satisfactory condition was achieved, and the left lower lobe was successfully excised under VATS, following which the bronchial stump was clamped using 2 titanium clips. Peak airway pressure was maintained at 30 cm H_2_O under manual ventilation in order to verify the safety and integrity of the bronchial stump. An obvious air leakage and continuous air bubbling were observed in the left cavity under manual ventilation. Unfortunately, we were unable to mechanically ventilate the lung due to the extent of air leakage. Manual ventilation was then performed for the right lung. As no air leakage was observed, these findings confirmed left lung leakage. To identify the site of leakage, the surgeon further mobilized the tracheobronchial tree. An irregular, circular, horizontal, and full-thickness left main bronchus rupture (1.2 cm) was observed at the tip of the bronchial tube, although the bronchial balloon remained intact.

The rupture was repaired via primary suturing with 4–0 prolene thread and secondary reinforcement with a pericardial flap through a left thoracotomy. The integrity of the repair was evaluated on the table. Following surgery, the double-lumen tube was removed, and the patient was re-intubated with a single-lumen tube (7.5 mm). The patient was transferred to the ICU for mechanical ventilation. Twenty-four hours postoperatively, his vital signs were stable. ICU physicians removed the tube after the patient had recovered the ability for spontaneous respiration. Repeated bronchoscopy revealed tight sutures and mild bronchial contraction 3 and 10 days following surgery, respectively. The patient was discharged 15 days after surgery and remained in good condition at the 2-month follow-up.

The patient provided informed consent for the publication of his clinical data. Medical Ethical Committee approval of the report was waived by Shaoxing People's Hospital (Shaoxing Hospital of Zhejiang University).

## 3 Discussion

Double-lumen tubes are widely utilized for isolated lung ventilation in thoracic practice. Improper placement of the double-lumen tube is among the most frequent causes of perioperative tracheobronchial injury during thoracic surgery.^[7]^ Causes for bronchial rupture during double-lumen tube intubation can be divided into 2 categories: technological and anatomic. Anatomic causes include congenital anomalies of the airway, large tumors invading or distorting the tracheobronchial tree, inflammatory lesions, and COPD. Bronchial rupture due to repeated surgical manipulation during double-lumen tube placement is a relatively rare complication whose mechanism remains undetermined. However, some previous evidence suggests that surgical intervention may impact inflammatory lesions and contribute to bronchial rupture.^[8]^

In the present case, a left-sided 37-French, Robershaw double-lumen tube was used for left lower lobectomy, and the tube was placed smoothly and correctly, in accordance with standard operating procedures. Moreover, the position of the tube was reconfirmed via FOB. These findings suggest that trauma associated with intubation had little effect on the resultant bronchial rupture, as intubation-related airway injuries usually occur longitudinal to the direction of tube insertion.^[7]^ In our patient, the nondeflated left lung impaired surgical visibility and access. To improve surgical conditions, carbon dioxide was insufflated into the thoracic cavity to compress the nondeflated left lung. As this action had no effect, forceps were then used to oppress and torque the left lung. After several attempts, a relatively satisfactory condition was achieved. During this process, the lung endured traction, compression, and even lobe torsion. In older patients, COPD may lead to chronic inflammation of the airway endothelium, over-inflation of the lung, and eventual vulnerability of the bronchus. When exogenous forces are applied, the most vulnerable portion of the airway is at the greatest risk for injury. In the present case, an irregular, circular, horizontal, full-thickness rupture of 1.2 cm was observed at the tip of the bronchial tube in the left main bronchus upon examination of the bronchial stump. As the left main bronchus is covered by a layer of peribronchial tissue, air leakage is often not detected until the subcarinal lymphatic nodes have been removed, and the surrounding tissue has been dissected. However, in the present case, the rupture was identified when examining the integrity of the bronchial stump, potentially due to peribronchial tissue depletion and distention associated with COPD.^[9]^ In this regard, thoracic hyperextension may contribute to injury of the tracheobronchial tree.^[10]^

Factors that may increase the risk of injury during placement of the double-lumen tune include the following^[8]^: inappropriate tube size, which results in excessive cuff inflation or inadequate placement in the mainstem bronchial lumen; malposition of the tip of the tube; rapid cuff inflation; asymmetric cuff distension, extension of the tip into the airway wall, or contact with carinal hooks (e.g., Carlens or White tubes); use of adjuncts such as bronchial blockers or tube-exchangers; airway wall weakness due to tumor infiltration or infection; steroid treatment; and use of nitrous oxide. As our patient was undergoing treatment with inhaled steroids for emphysema, COPD, and chronic bronchitis, these factors may have predisposed him to bronchial rupture.

VATS is recommended for patients with COPD requiring surgery due to its minimal invasiveness and clear view.^[11]^ However, thoracoscopy is associated with an increased risk of tracheobronchial injury, as this technique requires extensive lung collapse for good visualization. The thoracic surgeon uses forceps to compress and torque the lobe, which may also increase the risk of left bronchial rupture. In addition, carbon dioxide insufflation may result in more rapid or complete lung collapse and excessive torsion on at-risk bronchial structures. In the patient of the present case, COPD and chronic bronchitis may have caused delays in airway epithelial wound repair and chronic airway inflammation, both of which increase the vulnerability of the bronchial tree to extrinsic strain.^[9]^ Furthermore, such procedures may result in delayed diagnosis of injury (especially minor injury) due to variations in the field of view and quality of the videoscopic image. For these reasons, if the lung is not fully collapsed after several attempts, patients with COPD and bronchitis should undergo thoracotomy via VATS. Surgical compression and torsion of the lobe should be performed with extreme caution during VATS in order to prevent small or large airway trauma and rupture in at-risk patients.

## 4 Conclusion

Our findings suggest that surgical manipulation was the most likely cause of bronchial rupture in the present case. Thus, caution should be exercised during compression and torsion of the pulmonary lobe when attempting to improve surgical access, especially in patients with COPD. Conversion to thoracotomy is recommended if other measures have been unsuccessful. Immediate repair is recommended in high-risk patients when thoracotomy has already been performed.
